# The Effect of Metabolic Syndrome on Alzheimer’s Disease: Physical Activity as a Preventive and Therapeutic Measure

**DOI:** 10.3390/brainsci16050465

**Published:** 2026-04-26

**Authors:** Eleazar Ramírez Hernández, Citlalli Netzahualcoyotzi, Gabriela Hurtado-Alvarado, José Luis Sánchez, Ali Pereyra Morales, David Arredondo-Zamarripa, Luis Fernando Hernández-Zimbrón, Dulce Papy-Garcia, Jorge Guevara, Natalia Gutiérrez Ponce, Wilton Gomez-Henao, Yonathan Garfias, Gustavo Ortiz Chavez, Edgar Zenteno

**Affiliations:** 1 Departamento de Bioquímica, Facultad de Medicina, Universidad Nacional Autónoma de México, Mexico City 04510, Mexico; sanchez@bq.unam.mx (J.L.S.); ali@bq.unam.mx (A.P.M.); jorge.guevara@facmed.unam.mx (J.G.); zero_erh@hotmail.com (N.G.P.); wiltongomez@gmail.com (W.G.-H.); ygarfias@bq.unam.mx (Y.G.); ortizcg@facmed.unam.mx (G.O.C.); 2 Departamento de Farmacología, Centro de Investigación y de Estudios Avanzados del Instituto Politécnico Nacional (Cinvestav), Mexico City 07360, Mexico; cnetza@cinvestav.mx; 3 Departamento de Anatomía, Facultad de Medicina, Universidad Nacional Autónoma de México, Mexico City 04510, Mexico; hual.g313@gmail.com; 4 Departamento de Farmacología, Facultad de Medicina, Universidad Nacional Autónoma de México, Mexico City 04510, Mexico; davidarredondozamarripa@gmail.com; 5 Laboratorio de Investigación Interdisciplinaria, Escuela Nacional de Estudios Superiores Unidad León, Universidad Nacional Autónoma de México, Leon 37684, Mexico; 6 Glycobiology, Cell Growth and Tissue Repair Research Unit, Université Paris Est Creteil, F-94010 Creteil, France; papy@u-pec.fr; 7 Laboratorio Internacional Gly-CRRET-UNAM, Universidad Nacional Autónoma de México, Mexico City 04510, Mexico

**Keywords:** Alzheimer’s disease, metabolic syndrome, obesity, hyperglycemia, insulin resistance, therapeutic, physical activity

## Abstract

**Highlights:**

**What are the main findings?**

- The high prevalence of metabolic syndrome in the population increases the risk of developing neurodegenerative diseases such as Alzheimer’s disease.
- Metabolic syndrome involves changes in O-GlcNAcylation, glycosylation, mitochondrial dysfunction, oxidative stress, chronic inflammation, synaptic problems, impaired autophagy, and blood–brain barrier (BBB) dysfunction.

**What are the implications of the main findings?**

- Physical activity in patients with metabolic syndrome reduces the risk of developing Alzheimer’s disease, which is linked to better prognosis and improved cognitive function.
- A relatively new perspective suggests that regular exercise is crucial for maintaining brain metabolism during aging and has been proposed as both a preventive and therapeutic intervention.

**Abstract:**

Epidemiological and clinical research on neurodegenerative diseases has shown that metabolic dysregulations increase the risk of developing Alzheimer’s Disease (AD). Many metabolic changes can be grouped into metabolic syndrome (MetS), which is defined as the presence of three or more risk factors, including insulin resistance, hyperglycemia, hypertension, central obesity, and dyslipidemia. These changes cause systemic effects that are crucial in triggering neuroinflammation and neurodegeneration, key factors in AD development. All these factors impair energy metabolism in peripheral tissues and the brain by decreasing glucose utilization, leading to alterations in O-GlcNAcylation, glycosylation, mitochondrial function, oxidative stress, chronic inflammation, synaptic dysfunction, autophagy impairment, and blood–brain barrier (BBB) dysfunction. However, these factors are modified and largely influenced by lifestyle choices. A newer perspective emphasizes that regular exercise is vital for maintaining brain metabolism as we age. Current evidence suggests that engaging in physical activity for individuals with metabolic syndrome reduces their risk of Alzheimer’s disease, enhances prognosis, and improves cognitive abilities. This review explores how metabolic syndrome relates to Alzheimer’s and highlights possible strategies for prevention and treatment.

## 1. Introduction

In recent decades, the aging of the world population has become a public health problem due to the various chronic degenerative diseases they face. One of the neurodegenerative diseases most closely associated with aging is Alzheimer’s Disease (AD), which accounts for over 80% of dementia cases worldwide, with an expected increase to 150 million by 2050 [1]. AD is a progressive and irreversible neurodegenerative disease characterized by cognitive impairment with other symptoms becoming more severe as the disease evolves, ultimately culminating in impaired expressive speech and loss of executive functions. Two AD classifications have been described that share many similarities, including clinical, biomarker, and pathological outcomes: (1) Early-onset familial AD, associated with mutations in Amyloid Precursor Protein (APP), presenilin 1 (PSEN1), and presenilin 2 (PSEN2) genes that develop symptoms before 65 years of age, with an incidence of 5% of the total reported cases for AD. (2) Late-onset AD occurs after the age of 65 and represents 95% of the total reported cases, and age is the most critical risk factor [2]

Currently, there is no cure for AD, and available treatments only manage symptoms. However, epidemiological and clinical research suggest that AD may be linked to modifiable risk factors. These include diabetes, insulin resistance, obesity, hypertension, and dyslipidemia, which have been shown to increase the risk of developing AD [3,4,5]. Collectively, these factors are called “metabolic syndrome” (MetS) and are modifiable lifestyle factors. All components of MetS induce systemic changes that play a key role in neurodegeneration and neuroinflammation, which are central to AD development [6]. Recently, physical activity has been proposed as a therapeutic strategy that affects all components of metabolic syndrome and, consequently, reduces the risk of developing Alzheimer’s disease. This review aims to highlight current knowledge of the main metabolic dysfunctions that contribute to AD development and to identify potential areas for preventive and/or therapeutic interventions through physical activity.

## 2. Alzheimer’s Disease and Its Relationship with Metabolic Syndrome

The pathophysiology of AD is characterized by the presence of two histopathological markers that can be observed by microscopy: (1) extracellular neuritic plaques (NPs) formed by the aggregation of amyloid-*β* (A*β*) peptide; (2) the formation of neurofibrillary tangles (NFTs) that are the product of hyperphosphorylation and aggregation of the Tau protein [7,8]. The aggregation of NPs and NFTs occurs mainly in the entorhinal cortex, hippocampus, temporal lobe, frontal cortex, and certain subcortical areas, brain structures closely related to learning and memory [1,2,9]. Several studies have demonstrated links between metabolic disorders, A*β* peptide aggregation, and Tau protein hyperphosphorylation [10]. MetS in adulthood increases the risk of developing AD, either gradually or through combined mechanisms [11,12,13].

The global incidence of MetS increases with age, involving around 20% of men and 16% of women under 40 years of age; 41% of men and 37% of women between 40 and 59 years; and 52% of men and 54% of women older than 60 years [14,15,16]. MetS is a cluster of metabolic dysregulations that include central obesity, insulin resistance (IR), hyperglycemia, hypertension, and atherogenic dyslipidemia [17]. Furthermore, other factors related to MetS include chronic inflammation, endothelial dysfunction, genetic predisposition, hypercoagulability, and chronic stress [14,18,19]. The diagnosis of MetS has been made by scientific societies through worldwide criteria (Table 1), among all of which we highlight those proposed by the World Health Organization (WHO) [20], the International Diabetes Federation (IDF) [21,22], the National Cholesterol Education Program-Adult Treatment Panel III (NCEP-ATPIII) [23], the American Heart Association (AHA) [24,25], the American Diabetes Association (ADA) [26,27] and the European Association for the Study of Diabetes (EASD) [28].

Although the definitions of MetS are quite similar, the diagnostic criteria can vary in their units of measurement. These differences arise because they are designed for a multiethnic population and because the biomarkers aim to accurately estimate the prevalence of MetS. Furthermore, ethnic diversity means that some populations are more prone to severe abnormalities than others, as reflected in the average biomarker values for metabolic syndrome diagnosis. Nevertheless, the diagnosis is validated by the presence of at least three of the five major risk factors, which suffices to confirm MetS [6].

**Figure 1:**
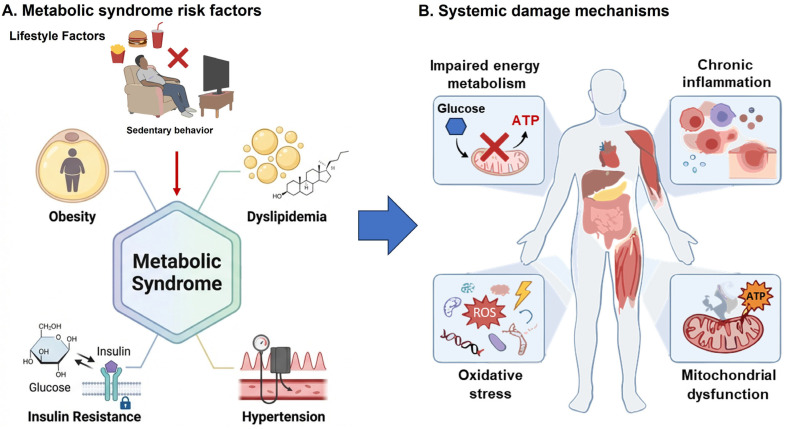
Metabolic syndrome is a risk factor for developing Alzheimer’s disease. (**A**) It is characterized by the coexistence of insulin resistance, obesity, hypertension, hyperlipidemia, and diabetes, often due to a sedentary lifestyle. (**B**) These factors contribute to systemic dysfunction marked by impaired cellular metabolism, mitochondrial dysfunction, oxidative stress, and chronic inflammation, all of which promote Alzheimer’s disease.

Research on the relationship between MetS and neurological disorders has yielded mixed results. However, studies in large populations have shown a higher prevalence of AD in patients with MetS. Middle-aged individuals with conditions such as diabetes, obesity, hypertension, hypercholesterolemia, or insulin resistance have an increased risk of developing dementia in adulthood. Furthermore, research in animal models has shown that rats and mice with metabolic syndrome induced by high-fat and high-sugar diets exhibit pathological changes like those seen in AD. These changes are associated with alterations in brain energy metabolism, including reduced glucose utilization, which affects processes such as *O*-GlcNAcylation, glycosylation, mitochondrial function, oxidative stress, chronic inflammation, synaptic dysfunction, impaired autophagy, and blood–brain barrier (BBB) permeability (Figure 1) [29,30,31].

**Figure 2:**
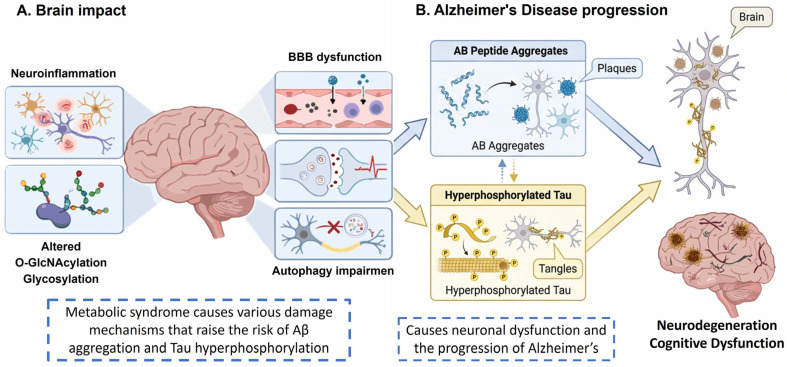
Correlation between metabolic disorders and the formation of PNs and NFTs. Effects on the brain include altered blood–brain barrier integrity, increased A*β* deposition, Tau hyperphosphorylation, neuroinflammation, autophagy alterations, and changes in glycosylation and O-GlcNAcylation that contribute to the progression of Alzheimer’s disease.

Additionally, there have been correlations between metabolic disorders and the formation of the NPs and NFTs (Figure 2). A*β* peptides are generated when APP is cleaved by β- and γ-secretase enzymes in the amyloidogenic pathway, which encourages NPs formation. Conversely, when APP is cut by α-secretase, A*β* production is inhibited, representing the non-amyloidogenic pathway [32,33]. Recent evidence indicates that sAPPα generated through the non-amyloidogenic pathway offers protective benefits for metabolic regulation and can boost membrane glutamate and glucose transporter activities in neurons. This has positive impacts on synaptogenesis, neurite growth, and neural progenitor cell proliferation [1]. Conversely, sAPPβ peptides produced by the amyloidogenic pathway have been linked to metabolic conditions such as obesity, type 2 diabetes mellitus (T2DM), non-alcoholic fatty liver disease, and cardiovascular disease. Additionally, the buildup of full-length APP causes mitochondrial dysfunction and lowers energy metabolism, effects that are observable in the brains of AD patients. Furthermore, in peripheral tissues, mitochondrial localization of APP in adipocytes can significantly impair mitochondrial function and promote obesity in mice, establishing a connection between obesity and AD [34,35]. In studies of AD in transgenic mice, the mechanisms of A*β* peptide toxicity, impaired glucose metabolism, and inflammation have been suggested to contribute to abnormal Tau hyperphosphorylation [36,37].

Tau protein becomes abnormally hyperphosphorylated, which promotes the formation of insoluble filaments that accumulate as NFTs. Additionally, the spread of Tau aggregates is linked to cognitive decline and reflects the progression of the disease [38]. Tau protein activity is controlled by various post-translational modifications (PTMs), such as phosphorylation, dephosphorylation, acetylation, ubiquitination, methylation, SUMOylation, glycation, glycosylation, O-GlcNAcylation, nitration, oxidation, and truncation (proteolytic cleavage) at serine, threonine, and tyrosine residues [39,40]. Disruptions in metabolism can impair Tau PTM regulation, alter protein properties, and lead to a loss of function. In AD brains, Tau from the frontal and parietal cortex showed modifications at 43–55 different phosphorylation sites, 19 acetylation sites, 14–17 ubiquitination sites, and 4 methylation sites [41,42,43]. The tendency for phosphorylation at T231, S235, and S262 appears to correlate with the clinical progression of AD and with the potential to induce local Tau accumulation [44]. In non-pathological conditions, phosphorylation of Tau depends on the balance between Tau kinases (GSK-3β, CdK5, PKA, MARK, Fyn, and others) and phosphatases (mainly PP2A); however, hyperphosphorylation of Tau is linked to dysfunction of these enzymes [38,45,46].

Moreover, hypertension damages blood vessel walls, leading to hypoperfusion, ischemia, and cerebral hypoxia, all of which contribute to AD development. Hyperlipidemia compromises the blood–brain barrier (BBB), increases A*β* accumulation, promotes Tau hyperphosphorylation, and induces neuroinflammation like that observed in AD. The role of obesity as an AD risk factor is still debated, but some studies indicate that midlife obesity raises dementia risk [10,13,29]. Mechanisms linking T2DM to AD include insulin resistance and impaired insulin signaling, among others. This raises important questions: Are metabolic dysfunctions directly connected to amyloid and Tau pathology? How do these pathological processes interact?

## 3. Impact of Insulin Resistance and Diabetes Mellitus on Alzheimer’s Disease

Insulin is a hormone produced by pancreatic β cells and released in response to rising plasma glucose levels after meals. It plays a crucial role in allowing glucose to enter adipose and muscle tissues, inhibits the release of excess free fatty acids from adipose tissue, and supports glucose production in the liver [47]. However, when tissues become less responsive, they develop insulin resistance, and the body compensates by producing more insulin to keep blood glucose levels normal [48,49]. In metabolic syndrome (MetS), insulin resistance is a key feature, negatively affecting both peripheral organs and the central nervous system (CNS). Excess insulin can cross the BBB and is broken down by the insulin-degrading enzyme (IDE), which also degrades A*β* peptides. During hyperinsulinemia, insulin competes with A*β* for IDE activity, leading to A*β* buildup. These findings suggest a significant overlap between the biological processes involved in both diabetes and Alzheimer’s disease [50,51].

Moreover, brain insulin resistance leads to decreased neurogenesis, neuronal plasticity, and cognitive decline by promoting neurodegeneration, changing dendritic spine density, and disrupting neurotransmission. These changes are seen in diseases such as obesity, diabetes, and AD. In the resistant state, altered insulin-evoked activity occurs in the hypothalamus, cortex, hippocampus, amygdala, cerebellum, striatum, and midbrain [10,52]. Patients with T2DM often show hyperinsulinemia and low insulin sensitivity, and epidemiological studies have found a link between T2DM and a higher risk of developing AD [10,53]. Proposed mechanisms for this connection include insulin deficiency, insulin resistance, impairment of the insulin receptor, hyperglycemia-induced toxicity related to advanced glycation end products (AGEs), inflammation, and cerebrovascular damage. Additionally, carrying the apolipoprotein E4 (ApoE4) allele and having T2DM significantly increases the risk of developing AD by five times [3,10,29]. Nevertheless, some antidiabetic drugs have demonstrated beneficial effects in treating AD [52,54,55], and improving insulin signaling can increase mitochondrial biogenesis, thus enhancing cognition in neuronal disorders associated with T2DM [47,49].

Postmortem analysis of brains from patients with Alzheimer’s disease (AD) shows a reduction in insulin receptor expression and changes in its intracellular signaling, both of which have been linked to poor cognitive performance [11,55]. Furthermore, insulin deficiency increases neuronal vulnerability to metabolic stress [55,56,57], as indicated by decreased insulin signaling and increased Tau hyperphosphorylation in the brains of individuals with AD and T2DM [58,59,60]. Although there is strong evidence linking cerebral insulin resistance to AD, there are also significant scientific contradictions and paradoxical findings. Many studies link alterations in insulin signaling to cognitive impairment, but others present inconsistent results, and the relationship between peripheral and central insulin dysfunction is not always linear.

## 4. O-GlcNAcylation, Insulin Resistance, and Tau Hyperphosphorylation

O-GlcNAcylation (O-GlcNAc) is a dynamic form of glycosylation that involves the addition of the monosaccharide N-acetylglucosamine (GlcNAc) to Serine (Ser) or Threonine (Thr) residues in nuclear, cytoplasmic, mitochondrial, and transmembrane proteins. The enzymes responsible for these modifications are O-GlcNAc transferase (OGT) and O-GlcNAcase (OGA), whose expression and activity depend on Glucose availability and metabolism through the hexosamine biosynthesis pathway. O-GlcNAc plays crucial roles at the cellular level, so any changes in O-GlcNAc levels or in MetS conditions may impact its function. Therefore, it is proposed that O-GlcNAc acts as a unique metabolic signaling mechanism, enabling cells to detect and respond to stress and thereby influencing cell survival [61,62,63]. Disruption of O-GlcNAc homeostasis is associated with various metabolic diseases such as T2DM, cardiovascular issues, and neurodegenerative diseases [63]. The link between metabolic signals and protein activity via O-GlcNAcylation suggests the potential to develop targeted therapies to increase cellular resilience in disease states.

One of the key features of MetS is insulin resistance, which alters O-GlcNAcylation by altering the availability of the donor substrate (UDP-GlcNAc) and OGT and OGA levels [64,65]. These changes disrupt metabolic homeostasis via O-GlcNAc signaling in specific cells and tissues. One example is O-GlcNAcylation in response to hyperglycemia, which increases NFκB activity, promoting pro-inflammatory processes. However, the role of O-GlcNAcylation in inflammation remains controversial [66]. At the CNS level, one example is the Tau protein, which has at least 12 potential O-GlcNAcylation sites, most of which compete with phosphorylation [39]. In AD, abnormal Tau phosphorylation by kinases such as GSK3β can be influenced by insulin [40,43,45,67]. Therefore, insulin resistance triggers abnormal activation of GSK3β, leading to increased phosphorylation and accumulation of hyperphosphorylated Tau, as well as the formation of NFTs. NFT accumulation alters the cytoskeleton, neuronal networks, and axonal transport, contributing to the loss of synaptic connections and progressive neurodegeneration [68,69]. However, increased O-GlcNAcylation can reduce the accumulation of hyperphosphorylated Tau. Recent research shows that glucosamine increases protein O-GlcNAcylation, helping to prevent neuronal loss in neurological disorders [69,70,71,72]. These findings highlight the diverse functions of O-GlcNAcylation, which not only control metabolic pathways but also the progression of neurodegenerative diseases.

## 5. Obesity as a Risk Factor in Alzheimer’s Disease

The relationship between obesity and Alzheimer’s disease (AD) is complex; it has not yet been confirmed whether obesity directly increases the risk of developing it. However, several studies suggest that abdominal obesity is a risk factor for dementia, especially in people over 65 years old, although its effects can appear before old age. Neuroimaging studies in obese individuals have shown structural changes similar to those seen in AD, such as reduced gray matter volume. These findings connect cognitive impairment to changes in long-term potentiation (LTP), loss of synaptic plasticity, and brain atrophy [73,74,75].

Obesity results from excessive accumulation of body fat [58,76,77]. The buildup of perivascular adipose tissue alters blood vessels and reduces blood flow to the brain, leading to ischemic injury [78,79]. This process causes chronic, low-grade systemic inflammation that worsens oxidative stress and can lead to neurodegeneration [80,81,82]. Certain brain areas are especially vulnerable to ischemia, including the CA1 and CA3 regions of the hippocampus, the caudate nucleus, cortical layers III, V, and VI, and the cerebellum [83,84]. Because of its high basal metabolic rate, the hippocampus is especially sensitive to oxygen and glucose deprivation, a factor believed to be a key contributor to memory loss [85,86].

Previous research suggests this vulnerability may be linked to leptin and insulin resistance, as well as chronic inflammation. Normally, leptin levels increase after eating to suppress appetite; however, in obesity, despite elevated leptin levels, satiety signals are impaired due to peripheral and central resistance [34,87,88]. High levels in adipose tissue of obese individuals act on the CNS, mainly affecting the hypothalamus, cerebral cortex, and hippocampus [88,89]. These regions harbor the long form of the leptin receptor (LepRb), the only variant capable of full signaling and most vulnerable to neuronal damage in AD [89,90,91]. Additionally, obesity can alter the APOE genotype and impact lipid metabolism and cognitive function. Evidence also suggests this impairment may be reversible; interventions targeting weight loss could restore or improve cognitive function in this group [92,93].

Recently, the gut microbiome and its interaction with the CNS have become key areas of research in obesity. The microbiome composition changes with diets high in fat and sugar, which can destabilize the gut–brain axis and increase AD risk [94,95]. These microbiome changes, called dysbiosis, can lead to neurodegeneration by disrupting peripheral neurotransmitter, metabolite, and immune signaling regulation [96,97]. Studies show that microbial diversity is significantly reduced in patients with AD, with decreases in Firmicutes and increases in Bacteroidetes at the phylum level, which correlates with greater cognitive decline [98,99]. This dysbiosis worsens pro-inflammatory signals through the gut–brain axis, harming health and triggering immune responses that, instead of restoring balance, can worsen systemic inflammation. Current research indicates that prebiotics and probiotics might help correct this imbalance, aiding in restoring homeostasis and possibly lowering AD risk [97,100].

## 6. Dyslipidemia

Dyslipidemia is usually characterized by high triglyceride (TG) levels in the blood, low levels of HDL cholesterol, and increased low-density lipoprotein (LDL) cholesterol [101]. Insulin resistance is a major abnormality that causes dyslipidemia and worsens other conditions, especially hypertriglyceridemia [102]. Elevated cholesterol levels, particularly LDL cholesterol, are well-known risk factors for coronary artery disease and stroke. Human epidemiological studies also show that high serum total cholesterol is linked to a greater risk of developing AD. This suggests that treatments for hyperlipidemias, such as statins, could help prevent AD [103,104].

Genetic studies of AD have identified single-nucleotide polymorphisms (SNPs) in several genes involved in cholesterol metabolism or transport, including Apolipoprotein E (APOE), Apolipoprotein J (APOJ/CLU), ATP-binding cassette subfamily A member 7 (ABCA7), and Sortilin-related receptor (SORL1) [104,105]. The APOE gene has three common variants: ε2, ε3, and ε4. Carrying specific polymorphisms in one of the ε4 alleles increases the risk by 12 to 15 times and lowers the age at which AD begins by about 15 years. The APOEε4 allele is also associated with higher plasma total and LDL cholesterol levels and promotes fibril formation from soluble A*β* [106]. Variations in the SORL1 gene, also called LR11, contribute to AD through multiple pathways. It is a key regulator of APP trafficking and processing, involved in A*β* breakdown, and interacts with ApoE and Tau [104,107]. The clusterin gene (CLU; also known as APOJ) plays a role in reverse cholesterol transport within HDL particles and affects A*β* peptide aggregation, promoting amyloid plaque formation. It may also help transport A*β* across the blood–brain barrier (BBB) and facilitate its uptake by glial cells and brain macrophages [108].

High cholesterol levels can disrupt the structure and function of the BBB, increasing the risk of AD [103,109,110]. An imbalance in lipid levels promotes the conversion of systemic cholesterol to 27-hydroxycholesterol, which can cross the BBB and promote the accumulation of A*β* peptides and Tau protein—hallmarks of AD [111]. Cholesterol has been shown to enhance the activity of β- and γ-secretase enzymes in the amyloidogenic pathway, increasing A*β* production from APP while reducing activity in the non-amyloidogenic α-secretase pathway. As a result, it accelerates A*β* buildup and influences other AD-related factors, including Tau hyperphosphorylation, neuroinflammation, cognitive decline, and dysfunction of cholinergic neurons [35,112,113].

Observational studies suggest that statins, which are commonly used to lower blood cholesterol, may decrease the incidence of AD or slow its progression [103,113,114]. However, clinical trials have not conclusively proven the effectiveness of statins in preventing or treating AD at different stages. Persistently high levels of free fatty acids can also be harmful, leading to low-grade inflammation that may cause insulin resistance [114,115]. Although fatty acids have limited ability to cross the BBB, PET studies demonstrate that they are taken up by the brain. Consequently, metabolic syndrome (MetS) can lead to fatty acid accumulation in the brain, which can be reversed by weight loss [116]. Experimental data indicate that high-fat diets can promote AD development, while diets rich in polyunsaturated fatty acids, such as docosahexaenoic acid, might offer protective effects [117]. Saturated fatty acids may promote brain inflammation by activating Toll-like receptor 4 (TLR4), with TLR4 loss-of-function protecting against the harmful effects of a high-fat diet [118,119]. The connection between high fatty acid levels and AD may involve A*β* and Tau, as free fatty acids have been shown to stimulate the formation of A*β* and Tau filaments in vitro, leading to cognitive impairment [113,114].

## 7. Inflammation

Metabolic inflammation resulting from MetS has been shown to impact the CNS, especially the hypothalamus [120,121]. The hypothalamus serves as a critical regulator of energy balance and body weight, and its dysfunction plays a role in obesity development [122,123]. A diet rich in saturated fatty acids can promote leptin and insulin resistance through the NF-κB pathway, limiting the hypothalamus’s capacity to control hunger and blood glucose levels [124]. Over time, these disruptions cause weight changes due to increased caloric consumption, leading to a chronic energy imbalance. Additionally, there is a reduction in synapse numbers within hypothalamic neurons and a rise in neuronal apoptosis [55,89,90].

The local and systemic inflammation induced by MetS can lead to disruption of the blood–brain barrier (BBB), with increased permeability and greater infiltration of immune cells. This results in sustained activation of glial and neuronal cells, causing hormonal dysregulation, increased immune sensitivity, or cognitive impairment, depending on the affected brain region [125]. Recently, transcriptome analysis has revealed that several AD risk genes are expressed in microglia. ApoE and the triggering receptor expressed in myeloid cells 2 (TREM2) have emerged as two of the main genes involved in both microglial activation and AD pathogenesis [126,127]. The TREM2 receptor is necessary for microglial proliferation, survival, and aggregation, which has been implicated in the phagocytosis of A*β* plaques and dead neurons with damaged myelin [128,129,130]. Variants of TREM2, such as R47H, R62H, and D87N, decrease TREM2 binding to ApoE, alter its binding to phospholipids, and increase the risk of sporadic AD. Notably, the R47H variant of TREM2 increases the risk of developing AD by 2 to 4 times, similar to the risk associated with carrying the ApoE ε4 allele [131,132]. Taken together, these findings suggest that microglia play a crucial role in susceptibility to metabolic disorders, which in turn contribute to the development of AD, rather than merely being a consequence of the disease process.

In AD, it has been observed that A*β* peptide aggregates can bind to different pattern recognition receptors (PRRs) expressed on microglia and astrocytes. One of the main receptors is TLR4, which recognizes pathogen-associated molecular patterns (PAMPs) and damage-associated molecular patterns (DAMPs), activating myeloid differentiation primary response 88 (MyD88), which in turn activates NF-κB [125,133]. Furthermore, binding of the A*β* peptide to the CD36 scavenger receptor and TLR4 leads to overactivation of microglia, resulting in a sustained inflammatory response and increased production of reactive oxygen species (ROS) [134,135]. ROS result from increased aerobic respiration in mitochondria, elevated NADPH oxidase activity in phagocytic cells, and nitric oxide (NO) production by inducible nitric oxide synthase (iNOS) [135]. This underscores the critical role of inflammatory cascades in the neurodegenerative process.

## 8. Hypertension

Although the brain accounts for only about a quarter of our body weight, it receives nearly 20% of our cardiac output. This elevated blood flow is essential due to its high metabolic demands, which, along with physiological systems, work together to regulate and support blood flow for brain activity. Consequently, circulatory disorders often have a notable impact on brain function [110,136]. Hypertension, specifically, can cause neurovascular problems and damage small cerebral arteries, arterioles, and capillaries, potentially leading to neuronal injury, synaptic dysfunction, and neurodegeneration, either alone or in combination with other factors [137].

Research indicates that middle-aged individuals with hypertension are at risk of developing AD and vascular dementia 15 to 20 years later. A study with over 300 participants found that hypertension can decrease cortical thickness and may promote AD via a pathway especially relevant for those carrying the APOE4 gene variant. Hypertension damages blood vessel walls, leading to hypoperfusion, ischemia, and cerebral hypoxia [136,138,139]. Endothelial injury activates astrocytes and microglia, sparking inflammatory responses and releasing vasoactive cytokines and chemokines. This degeneration results in a loss of capillary dilation in response to neuronal signals, causing hypoperfusion and BBB breakdown, promoting the buildup of blood-derived toxins and fluid in the perivascular space. Emerging evidence suggests that blood vessel damage can trigger a cascade leading to A*β* accumulation and increased NFTs in the hippocampus [140,141]. Lifestyle choices, such as moderate exercise and a healthy diet, can positively influence these effects by supporting cardiovascular and cerebrovascular health.

## 9. AGES and RAGES

When sugars react with the amino groups of lysine side chains in proteins, they generate advanced glycation end products (AGEs), which can bind to their ligands on the RAGE receptor in cells [142,143]. AGEs and RAGE increase under hyperglycemic conditions, such as in diabetes, leading to chronic inflammation, oxidative stress, and vascular complications. High blood glucose levels lead to the accumulation of AGEs, which bind to RAGE and cause cellular damage and aging, influencing the progression of these diseases. Glycoxidation is particularly relevant to AD because extracellular fibrillar aggregates of A*β* have similar characteristics to AGEs and bind to RAGE receptors on neurons and brain endothelial cells [144,145]. This interaction increases oxidative stress, leading to neuronal death and vascular dementia in AD. Furthermore, glycation can keep the A*β* peptide in oligomer form, accelerating vascular dysfunction and worsening cognitive decline in AD [142,145].

## 10. Physical Activity and Contribution to the Prevention of Metabolic Syndrome and Alzheimer’s Disease

**Figure 3:**
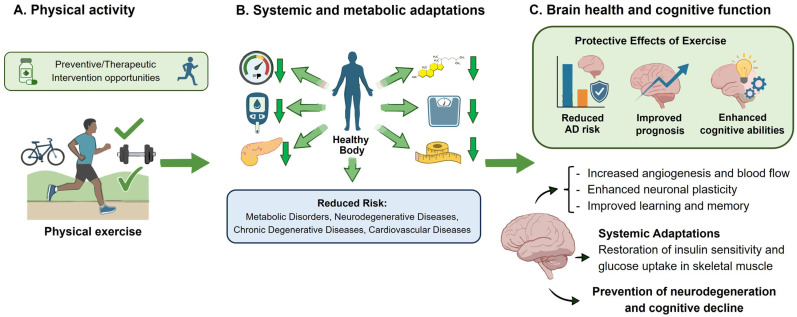
Lifestyle changes and increased physical activity can help prevent the start and progression of Alzheimer’s disease. (**A**) Engaging in physical exercise acts as a preventive or therapeutic measure that supports brain health. (**B**) This benefit comes from systemic metabolic adaptations triggered by the activity. (**C**) Generally, such habits lower the risk of developing AD, improve cognitive functions, and promote the release of neurotrophic factors crucial for processes like angiogenesis, neurogenesis, neuronal plasticity, and enhanced learning and memory.

The benefits of physical activity are widely recognized and crucial for maintaining the body’s balance. Lifestyle changes and exercise are vital for good health, helping to prevent many chronic illnesses today [146,147,148]. Exercise induces long-term adaptations that lower cardiovascular risk factors, blood pressure, Glucose, Insulin, total Cholesterol, body mass index, and waist circumference. As a result, physical activity is a promising approach for alleviating various diseases, including metabolic disorders, neurodegenerative conditions, chronic degenerative diseases, many types of cancers, and cardiovascular diseases (Figure 3) [149,150].

Physical activity is categorized into two types: (1) Endurance (aerobic) exercise, which involves glucose metabolism that relies on oxygen under aerobic conditions; (2) Resistance (anaerobic) exercise, which involves weight or overload training in anaerobic conditions and involves short bursts of high- or maximum-intensity activity [151,152]. Different types of exercise provoke diverse physiological adaptations in cardiovascular and respiratory systems, while skeletal muscle movement determines the body’s functional capacity, performance, and health outcomes [151].

Although much focus has been placed on skeletal muscle adaptations, all organs are impacted both immediately and over time. Primary peripheral tissues secrete various proteins, such as myokines from skeletal muscle, hepatokines from the liver, and adipokines from fat tissue. These proteins help regulate energy balance and increase insulin sensitivity. During exercise, some of these proteins, called “exerkines,” are secreted and create a complex inter-organ network that supports the systemic metabolic benefits of exercise [153,154]. Bioactive myokines are released from contracting skeletal muscles during exercise, along with numerous cytokines, peptides, and metabolites [155,156,157]. Myokines may mediate many systemic benefits, influencing adipose tissue, liver, pancreas, heart, blood vessels, and brain, thus aiding in protection against neurodegeneration.

Exercise also affects cognitive function and brain health, which is especially relevant for healthy aging. The secretory response of skeletal muscle encourages the release of brain-derived neurotrophic factor (BDNF), which promotes neuron growth; vascular endothelial growth factor (VEGF), which supports the development of vital blood vessels; and insulin-like growth factor (IGF-1), essential for exercise-induced blood vessel growth (angiogenesis) [157,158]. Consequently, exercise is well-documented to increase angiogenesis and blood flow in the brain [159]. Because brain metabolism depends on oxygen and glucose, expanding capillary networks improves their supply, supporting neuronal plasticity. This plasticity involves forming new synapses and growing neuronal networks. These structural and functional improvements enhance learning and memory, resulting in more efficient cognitive function. As we age, maintaining a brain capable of better neural plasticity or function becomes increasingly critical. Older adults in good physical health tend to have greater cognitive reserves and experience slower aging. Even amid cognitive decline, individuals can improve cognition through exercise as a therapeutic strategy [156,159]. Therefore, exercise has been shown to significantly enhance cognition and mood, especially in patients with AD.

All these factors influence long-term brain health, and regular physical activity boosts brain function regardless of health status or age. Additionally, exercise promotes better overall fat oxidation and reduces visceral fat. For instance, impaired insulin signaling, often called insulin resistance, is regarded as a common feature of MetS, with harmful effects both peripherally and in the central nervous system. Brain insulin resistance is linked to conditions such as obesity, diabetes, and AD, which are also mainly associated with altered metabolic function. Insulin acts on neural circuits to regulate systemic metabolism and body weight by activating its receptors in neurons and other cells. Disruption of insulin receptors in the brain leads to decreased neuronal plasticity and cognitive decline. Insulin deficiency promotes neurodegeneration, as it is closely linked to maintaining neuronal plasticity, dendritic spine density, neurotransmission, and even adult neurogenesis. In insulin-resistant states, abnormal insulin-evoked activity is observed in regions like the hypothalamus, cortex, hippocampus, amygdala, cerebellum, striatum, and midbrain. Compromised insulin sensitivity can be restored through exercise, which enhances skeletal muscle glucose uptake and improves blood sugar control [55,56].

The neural basis of exercise-based therapeutic interventions has been thoroughly mapped in animal models. These studies indicate that physical activity or exercise can induce positive morphological and functional changes in the brains of older animals. Exercise interventions stimulate changes in the motor cortex, cerebellum, striatum, and hippocampus in rats. Aerobic exercise boosts functional connectivity and increases gray and white matter volumes in the motor, prefrontal, and temporal cortices. Although previous research has shown the potential for exercise to enhance brain plasticity, the specific functional changes in brain regions that explain the improvements seen in middle-aged and older adults remain unclear. Protective mechanisms may involve promoting metabolic stress through exercise, resulting in long-term adaptations that safeguard against metabolic and neurodegenerative diseases. However, the exact mechanisms by which exercise protects the CNS are still debated. Therefore, the relationship between exercise and brain health is complex and likely depends on the adult life stage [157]. Multiple mechanisms could explain how exercise benefits the brain and potentially delay Alzheimer’s disease progression. In mice, physical activity enhances vascular health and boosts neurotrophic factors in the brain, which promote neurogenesis, support neuronal survival, and facilitate the creation of new synaptic connections.

## 11. Exercise as a Therapeutic Strategy in Alzheimer’s Disease

Related to aging-related comorbidities caused by insulin signaling deficiency [160], insulin resistance may work together with inflammation, oxidative stress, excess fat in adipose tissue, and the accumulation of lipids in the liver and muscles, as well as the loss of lean muscle mass that can happen with aging, to increase the risk of T2DM and fatty liver [161,162]. Physical activity might serve as a strategy to boost cognitive function or at least slow cognitive decline in individuals at risk of AD and affected patients [152,160]. Consequently, multimodal interventions that include adopting an active lifestyle should be recommended for older adults [163,164]. The availability of transgenic mouse models that replicate key neuropathological features of AD has allowed studying the main protective effects of exercise on brain aging. Studies by Choi et al. demonstrate that, in a 5xFAD transgenic mouse model of AD, exercise enhances memory by promoting hippocampal neurogenesis and increasing brain-derived neurotrophic factor (BDNF) levels. This research is significant because it shows that stimuli boosting BDNF expression and neurogenesis can effectively prevent or slow AD progression or contribute to its mitigation. Moving forward, understanding how exercise influences neurogenesis and BDNF levels in the CNS, especially at the synapse, cellular (neurons, glia, and vascular cells), and circuit levels, will be crucial [165]. In fact, it is well established that exercise encourages the formation of new synapses and benefits cardiovascular health, both of which are relevant for treating MetS and AD [139,149,157,166]. If these findings are replicated in other models and are relevant to humans, this research indicates that we might be able to harness the effects of exercise to improve the prevention and/or treatment of dementia.

Studies by van Praag et al. (2000) have shown that an “enriched environment,” which involves placing animals in large cages with running wheels, colorful tunnels, and assorted toys, can promote hippocampal neurogenesis and neuronal plasticity [167]. Thus, animals placed in an enriched environment improve memory formation and increase neuronal proliferation, differentiation, and integration in the dentate gyrus of the hippocampus, as well as recover from memory deficits induced by injury [168]. Morphologically, exposure to an enriched environment increases the number of dendritic spines, ramifications, and formed synapses, suggesting these changes result from the context and level of the stimulus [168,169]. This aligns with studies showing that older adults who exercise are more likely to maintain cognitive function [170]. Physical activity promotes A*β* turnover, reduces inflammation, and enhances neurotrophin synthesis and release, while improving cerebral blood flow, thereby offering brain protection. Changing lifestyle habits during the presymptomatic and pre- dementia stages in the elderly may positively delay the development of metabolic disorders and dementias [164,171].

In the early stages of AD, cognitive functions—particularly episodic memory—are impaired, with the hippocampus playing a crucial role [9,172,173]. However, it remains unclear whether the cognitive deficits stem from alterations in encoding and consolidation of episodic information or from impaired retrieval of stored memories. During early AD, changes in synaptic phenotypes have been identified as the main correlations of cognitive deficits in both human patients and mouse models [172]. The deposition of the A*β* peptide and the formation of amyloid plaques lead to a progressive reduction in dendritic spine density in neurons within the hippocampal memory engram. Studies with transgenic mice in early AD stages show that direct optogenetic activation of neurons forming the hippocampal memory engram results in recovery of dendritic spines and long-term memory. This demonstrates that although transgenic mice exhibit amnesia, optogenetic induction of long-term potentiation at engram neuron synapses restores dendritic spine density and long-term memory [174]. Therefore, selectively salvaging dendritic spines in engram neurons may be an effective strategy to treat memory loss in early AD. One benefit of physical activity is that it extends beyond skeletal muscle, inducing adaptations in other organs. In summary, physical exercise promotes functional changes in hemodynamics, synaptic plasticity, neurogenesis, and neural cell proliferation, leading to the formation of new neurons that are functionally integrated into neural networks.

## 12. Conclusions

This manuscript explores metabolic alterations in MetS to better understand their impact on dementia, particularly AD. It highlights mechanisms such as obesity, hypertension, dyslipidemia, inflammation, and T2DM. Additionally, it suggests strategies to prevent or slow neurodegenerative diseases. With no effective treatments currently available for AD, managing modifiable risk factors like obesity, diabetes, hypertension, and dyslipidemia (metabolic syndrome) is essential to delay disease onset and progression, thereby improving patients’ quality of life. While T2DM has garnered much attention, targeting neuronal insulin signaling remains controversial regarding its role in AD development. Since current drugs do not alter the disease’s fundamental mechanisms, research is increasingly focusing on other brain cells—microglia and astrocytes—and the most affected neurons. Understanding how lifestyle influences these cellular interactions and identifying specific biological molecules will be key to developing more effective prevention and treatment strategies.

## Figures and Tables

**Table 1:** Diagnostic criteria for metabolic syndrome.

Criteria/Clinical Measure	WHO (1998)	NCEP-ATPIII (2001)	IDF (2005)	AHA/NHLBI (2009)
Central Obesity	Waist/hip ratio Men: >0.9 Women: >0.85 BMI > 30 Kg/m^2^	Waist Circumference Men: >40″ Women: >35″	Waist Circumference Men: >37″ Women: >32″	Waist Circumference Men: >40″ Women: >35″
Blood Glucose	Fasting glucose (≥110 mg/dL) Post-load glucose at 2 h (≥140 mg/dL and 200 mg/dL) Glucose intolerance Insulin resistance	Diagnosis of type 2 diabetes Fasting glucose (≥110 mg/dL) Glucose intolerance Insulin resistance		
High triglycerides	≥150 mg/dL			
Low HDL	<35 mg/dL in men <39 mg/dL in women	Men: <40 mg/dL Women: <50 mg/dL		
High Blood Pressure	≥140/90 mmHg	≥130/85 mmHg		
Diagnosis	≥3 criteria one of which should be insulin resistance	≥3 criteria	≥3 criteria one of which should be central obesity	≥3 criteria

The criteria for the diagnosis of metabolic syndrome were established by the World Health Organization (WHO) in 1998, National Cholesterol Education Program-Adult Treatment Panel III (NCEP-ATPIII). Similar criteria were updated in 2006 by the International Diabetes Federation (IDF) and American Heart Association (AHA). Metabolic syndrome is diagnosed if at least three of the following criteria are met. BMI: body mass index; HDL-Cholesterol: High-density lipoproteins.

## Data Availability

No new data were created or analyzed in this study. Data sharing is not applicable to this article.
